# Comorbidities Associated with Vitiligo: Results from the EpiChron Cohort

**DOI:** 10.3390/jcm14020432

**Published:** 2025-01-11

**Authors:** Beatriz Clemente Hernández, Itziar Muelas Rives, Tamara Gracia Cazaña, Marcial Álvarez Salafranca, Beatriz Poblador-Plou, Clara Laguna-Berna, Aida Moreno Juste, Antonio Gimeno-Miguel, Yolanda Gilaberte

**Affiliations:** 1 Department of Dermatology, Miguel Servet University Hospital IIS Aragon, 50009 Zaragoza, Spain; itziar.muelas.rives@gmail.com (I.M.R.); tamgracaz@gmail.com (T.G.C.); marcialaspn@gmail.com (M.Á.S.); ygilaberte@gmail.com (Y.G.); 2 Research Group of the Government of Aragon B59_23D Dermatology and Photobiology, Aragon Health, Miguel Servet University Hospital, 50009 Zaragoza, Spain; 3 EpiChron Research Group, Aragon Health Sciences Institute (IACS), Aragon Health Research Institute (IIS Aragón), Miguel Servet University Hospital, 50009 Zaragoza, Spain; bpoblador.iacs@aragon.es (B.P.-P.); clagunab.iacs@aragon.es (C.L.-B.); aidamorenoj@gmail.com (A.M.J.); agimenomi.iacs@aragon.es (A.G.-M.); 4 Research Network on Chronicity, Primary Care, and Health Promotion (RICAPPS), Institute of Health Carlos III (ISCIII), 28029 Madrid, Spain; 5 Aragon Health Service (SALUD), 50003 Zaragoza, Spain

**Keywords:** vitiligo, comorbidities, epidemiology, multimorbidity, population-based cohort, autoimmune diseases

## Abstract

**Background:** Vitiligo is a pigmentation disorder that impacts approximately 0.5% to 2% of the global population. Growing interest surrounds the comorbidities associated with vitiligo. This study aimed to describe the socio-demographic characteristics of the patients with vitiligo in Aragón (Spain) and to investigate their associated comorbidities. **Methods:** A retrospective observational study was conducted using clinical data from individuals in the EpiChron Cohort (reference population of 1.3 million) who were diagnosed with vitiligo between 1 January and 31 December 2019. The prevalence of chronic comorbidities was calculated using logistic regression models, obtaining the odds ratio (OR) of each comorbidity (dependent variable) according to the presence or absence of vitiligo (independent variable). We used a cut-off point for a statistical significance of *p*-value < 0.05. **Results:** In total, 218 patients diagnosed with vitiligo were analyzed. The mean age was 44.0 years, and 56.42% were female. The largest proportion of patients (34.86%) were aged between 18 and 44 years. Among all vitiligo patients included, 71.5% presented multimorbidity, with an average of 3.21 diagnosed comorbidities. The conditions most frequently associated with vitiligo included thyroid disorders (OR: 3.01, *p* < 0.001), ocular and hearing abnormalities (OR: 1.54, *p* < 0.020), inflammatory skin disorders (OR: 2.21, *p* < 0.001), connective tissue diseases (OR: 1.84, *p* < 0.007), lower respiratory tract diseases (OR: 1.78, *p* < 0.014), urinary tract infections (OR: 1.69, *p* < 0.032), and cardiac arrhythmias (OR 1.84, *p* < 0.034). **Conclusions:** This research highlights the importance of understanding the broader health implications of vitiligo and provides a foundation for further exploration into the complex interplay between this dermatologic condition and a diverse range of comorbidities.

## 1. Introduction

Vitiligo is a skin disorder characterized by the presence of depigmented patches on the skin due to the loss of melanin. It is estimated to affect about 0.5 to 2% of the population [1]. Vitiligo affects all age groups, with no predilection for gender or race. The variability in its prevalence seems to be influenced by genetic, environmental, and other factors that are not fully understood [2,3,4].

The international recommendations for the management of vitiligo provide an updated classification of the condition, reflecting a global consensus on nomenclature. It identifies three main categories: (1) non-segmental vitiligo (NSV) (term “vitiligo”), which includes subtypes such as acrofacial, mucosal (more than one site), generalized, universal, and mixed (associated with segmental vitiligo); (2) segmental vitiligo (SV), characterized by unilateral, bi-, or plurisegmental distribution; and (3) undetermined/unclassified vitiligo, encompassing variants such as focal vitiligo (localized lesions) and mucosal vitiligo limited to a single site [2,5]. SV, which accounts for only 10–20% of cases of vitiligo, usually presents at a younger age, and it is not as frequently associated with autoimmune disorders [2,3,5]. On the other hand, vitiligo (both VNS and VS) can be defined regarding its activity, focusing on the progression of the disease. Rapidly spreading/progressive vitiligo is defined as the emergence of numerous new lesions or significant enlargement of existing ones within the past three months, and stable vitiligo is characterized by the absence of significant changes in lesions for at least 12 months [5].

The exact mechanism by which melanocytes are lost in vitiligo has been the subject of debate and research for many years. Its pathogenesis involves a complex interaction of genetic, autoimmune, oxidative stress, and environmental factors. Genetically, susceptibility is linked to variants affecting immune regulation and melanocyte function. Immune mechanisms, particularly those involving cytotoxic CD8+ T cells, play a central role; these cells interact with melanocytes and drive disease progression through the local production of IFN-γ. This, in turn, triggers IFN-γ-induced chemokine secretion, attracting even more T cells to the skin through a positive feedback mechanism [6,7]. Disease relapse after the cessation of treatment is driven by autoreactive tissue-resident memory (TRM) cells [8]. The autoimmune theory is currently the most widely accepted. A high prevalence of autoantibodies against melanocytes and genes shared with other autoimmune diseases has been found in these patients, as well as a higher prevalence of autoimmune diseases among first-degree relatives [2,7,8,9,10]. On the other hand, oxidative stress exacerbates melanocyte vulnerability, and increased levels of reactive oxygen species (ROS) contribute to melanocyte damage [11]. Various environmental triggers, such as physical trauma (*Koebner phenomenon*), exposure to chemicals, heat, smoking, and certain medications and viral infections, further influence the onset or progression of the disease [6,12].

The diagnosis of vitiligo is primarily based on clinical examination, identifying characteristic white patches on the skin caused by the loss of melanocytes. According to the international recommendations for the management of vitiligo, it is necessary to distinguish between cases with uncertain and certain diagnoses. For uncertain cases, Wood’s lamp examination is advised to differentiate between hypochromic and depigmented skin, along with skin biopsies from lesional areas and additional tests, such as mycological or molecular assessments, depending on the differential diagnosis. For cases with a clear diagnosis, routine screening tests for anti-thyroid antibodies and thyroid function are recommended, with further tests to see if there are indications of autoimmune diseases [5,9].

A management algorithm for non-segmental vitiligo has been proposed based on disease activity, severity, and individual therapeutic objectives. This approach emphasizes shared decision making between the physician and the patient, considering topical treatments such as immunomodulators, targeted phototherapy with NB-UVB, and systemic therapies for rapidly progressing cases. For patients with stable disease, options like maintenance therapies to prevent relapses and, in selected cases, surgical techniques are recommended. For stable segmental vitiligo, surgical techniques such as grafting may be considered to promote repigmentation. In early stages or progressive cases, topical treatments with immunomodulators and targeted phototherapy with NB-UVB are recommended [5]. Emerging research in vitiligo treatment focuses on JAK inhibitors, which block inflammatory pathways involved in melanocyte destruction. *Ruxolitinib* cream, the first FDA- and EMA-approved treatment for non-segmental vitiligo, has shown promising results in stabilizing the disease and promoting repigmentation. Ongoing studies on other topical and systemic JAK inhibitors indicate a promising future for more effective and tailored therapies [5,13,14,15,16].

Various studies have explored the association between vitiligo and other conditions, including autoimmune, systemic, and dermatological diseases. Common comorbidities include thyroid disorders, type 1 diabetes, pernicious anemia, Addison’s disease, connective tissue disorders, ocular and hearing diseases, skin conditions (such as psoriasis, atopic dermatitis, and alopecia areata), and psychological and emotional impacts, potentially leading to depression, anxiety, and social isolation [3,10,17,18,19].

The presence of these vitiligo-associated conditions can vary among individuals, and the relationship between vitiligo and these comorbidities is complex and not fully understood. There remains a gap in knowledge about the specific comorbid conditions most commonly associated with vitiligo, particularly in different geographic regions and populations. This study, focused on patients in the Aragon region of Spain, aimed to fill this gap by describing the socio-demographic profile of vitiligo patients and analyzing the most prevalent comorbidities in this population.

## 2. Materials and Methods

### 2.1. Study Design and Population

We conducted a retrospective, observational study on the EpiChron Cohort, which links socio-demographic and clinical data from all the users of the public health system of the Spanish region of Aragon [20]. This cohort is based on the information registered in the electronic health records (EHRs) and clinical–administrative databases of approximately 98% of the citizens of the region (reference population: 1.3 million people). For this study, we selected all the individuals from the cohort with at least one chronic condition in 2019 as the reference population (i.e., 1,019,690 patients). Of them, we selected 218 individuals diagnosed with vitiligo at some point from 1 January 2019 to 31 December 2019 to study their comorbidity.

This study was approved by the Clinical Research Ethics Committee of Aragon (CEICA) (Research Protocol PI23/411) and waived the requirement to obtain informed consent from patients given the epidemiological nature of the project, which used anonymized data.

### 2.2. Variables and Data Sources

For all patients (with and without vitiligo), we studied socio-demographic variables and all chronic diseases registered in their EHRs. As socio-demographic variables, we included sex, age (categorized as 0–17, 18–44, 45–64, and ≥65 years), nationality (i.e., country of birth), area of residence (urban, i.e., people living in municipalities that concentrate at least 80% of the population of the area, and rural, i.e., the rest), and the deprivation index of the area. This index was developed for Aragon and calculated at an aggregated level with the basic healthcare area according to 26 socio-economic indicators (Table 1) including information on housing, education, neighborhood conditions, types of employment, unemployment rates, the aging of the population, and immigration, and it was divided into four quartiles from least (Q1) to most (Q4) deprived [21]. Finally, within the socio-demographic characteristics, we included the number of chronic diseases, as well as the presence or absence of multimorbidity (i.e., the presence of two or more chronic diseases) [22].

These diagnoses were initially categorized using the International Classification of Primary Care (ICPC-1) or the International Classification of Diseases, Ninth Revision, Clinical Modification (ICD-9-CM). To facilitate their management and interpretation, they were grouped into broader diagnostic categories. We utilized the freely available Clinical Classifications Software (CCS), version ICD-9-CM (Agency for Healthcare Research and Quality, Rockville, MD, USA) [23], which applies an open source algorithm to classify individual codes into 226 general categories based on clinical, therapeutic, and diagnostic similarities. Subsequently, 153 of these conditions were classified as chronic using the Chronic Condition Indicator (CCI) [24] open source tool, which defines chronic conditions as those lasting six months or longer, including past conditions that require ongoing management, significant risk of recurrence, or continuing implications for patient care.

### 2.3. Statistical Analysis

First, a descriptive analysis of the socio-demographic characteristics of the population with vitiligo was performed. We summarized the results as frequencies and proportions for categorical variables and as means and standard deviations for continuous variables. We conducted logistic regression models to estimate the likelihood of presenting vitiligo according to sex, age, nationality, area of residence, and the area deprivation index as crude and sex- and age-adjusted odds ratios (OR). For the analysis of vitiligo comorbidity, we described the frequency and prevalence of chronic diseases in the population with vitiligo. Then, for the identification of those comorbidities systematically associated with vitiligo, we used logistic regression models to calculate the occurrence risk of each comorbidity (dependent variable) according to the presence or absence of vitiligo (independent variable).

All analyses were conducted in STATA software (Version 12.0, StataCorp LLC, College Station, TX, USA), with the statistical significance set at *p* < 0.05. To increase the clinical interest of this study and to facilitate the interpretation of the results, only diseases with a prevalence > 1% were included in the analysis.

## 3. Results

### 3.1. Characteristics of the Population

**Figure 1:**
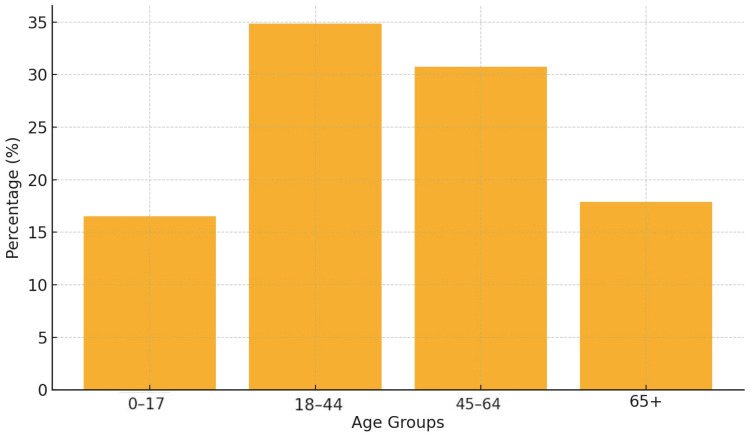
Age distribution of vitiligo patients.

We analyzed a population of 218 patients with vitiligo (56.4% women). The mean age was 44.0 years (SD 21.26), and regarding age groups, the highest percentage of patients (34.86%) fell within the 18–44 age group, accounting for 34.86% of the population. This is followed by the 45–64 group at 30.73%, highlighting the condition’s prevalence among adults. Patients aged 65 and older represented 17.89%, while those under 17 made up 16.51% of the cohort (Figure 1). The demographic characteristics are shown in Table 2.

Regarding the likelihood of presenting vitiligo based on sex, age, nationality, area of residence (urban/rural), and the deprivation index of the area, we found statistically significant differences (OR [95% confidence interval]) in nationality and age. People born in Latin America showed a 3.06 [2.07–4.53] times higher risk of presenting vitiligo compared to the Spanish population (*p* < 0.001), whereas people aged ≥65 years presented a lower likelihood (0.48 [0.30–0.76]) of presenting vitiligo compared to younger people aged 0–17 years (*p* = 0.002) (Table 3). No relevant differences in the prevalence of vitiligo according to the area of residence, socio-economic deprivation, and sex were observed.

### 3.2. Chronic Comorbidities

**Figure 2:**
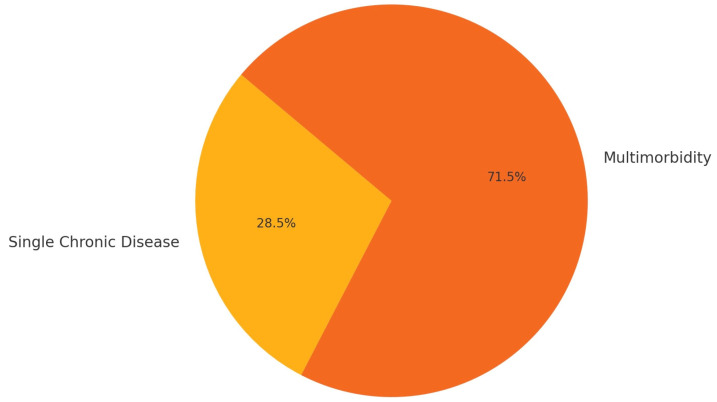
Proportion of multimorbidity among vitiligo patients.

Of all the patients with vitiligo included, 71.5% had multimorbidity, being diagnosed with a mean of 3.21 comorbidities (SD 2.41). This multimorbidity was more common in woman (76.42%) than in men (65.26%) (Figure 2) (Table 2).

**Figure 3:**
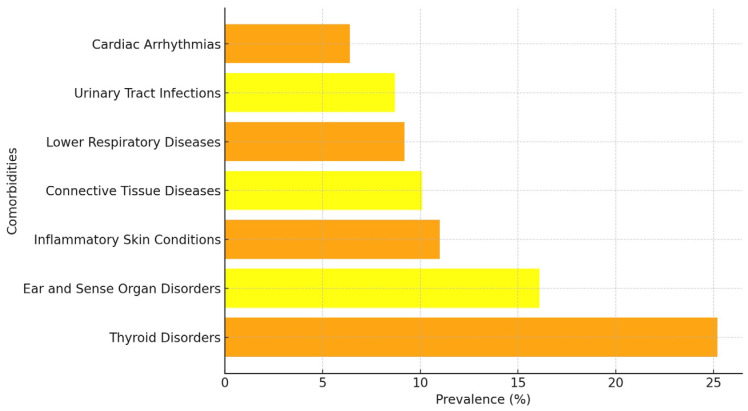
Prevalence of main comorbidities in vitiligo patients.

The most common chronic comorbidities in people with vitiligo of all ages and for both sexes were thyroid disorders (25.2%), ear and sense organ disorders (16.1%), inflammatory conditions of skin (11%), and connective tissue diseases (10.1%). All these comorbidities were more prevalent in the vitiligo population compared to the general one (Figure 3) (Table 4).

Regardless of their prevalence, after adjustment by sex and age, the conditions most associated with vitiligo were as follows (adjusted OR (95% CI)): thyroid disorders (3.01 [2.18–4.15], *p* < 0.001), followed by inflammatory conditions of skin (2.21 [1.45–3.38], *p* < 0.001), connective tissue diseases (1.84 [1.19–2.87], *p* = 0.007), cardiac dysrhythmias (1.84 [1.05–3.22], *p* = 0.034), lower respiratory diseases (1.78 [1.12–2.82], *p* = 0.014), urinary tract infections (1.69 [1.05–2.73], *p* = 0.032), and ear and sense organ disorders (1.54 [1.07–2.22], *p* = 0.020) (Table 4).

On the other hand, there were also some relevant comorbidities that did not show an association with vitiligo, including depression and mood disorders (0.88; [0.56–1.37], *p* = 0.563), anxiety (0.92; [0.62–1.36], *p* = 0.660), hypertension (1.12; [0.76–1.63], *p* = 0.569), disorders of lipid metabolism (1.11; [0.80–1.53], *p* = 0.532), and diabetes mellitus (1.39; [0.86–2.27], *p* = 0.181), among others (Table 4).

## 4. Discussion

Vitiligo is a pigmentary disorder that has been mostly considered a cosmetic disease for many years. Our study shows that the majority of these patients have multimorbidity. Thyroid diseases emerged as the most frequent comorbidities, followed by ocular and audiological disorders, within the vitiligo population in Aragon. Additionally, strong associations were observed with inflammatory skin diseases, connective tissue disorders, and certain non-dermatological conditions such as respiratory infections, urinary tract infections, and cardiac arrhythmias.

The present study analyzed the socio-demographic characteristics of the patients with vitiligo diagnosed in the region of Aragon. A study reviewing more than 50 studies worldwide estimated that vitiligo affects approximately 0.5–2% of the world’s population [1]. There is no racial predilection, and it affects adults and children of both sexes equally. NSV typically starts in early childhood or young adulthood, with the highest onset occurring between the ages of 10 and 30. Around 50% of patients develop vitiligo before the age of 20 and 70–80% before turning 30 [3,9]. The prevalence tends to decrease with increasing age. In this regard, our study shows that 65.6% of patients are aged between 18 and 64 years, with no gender predilection.

In our study, we found no significant differences between living in an urban or rural environment, in contrast to other inflammatory diseases such as atopic dermatitis, where such differences have been found [25]. In addition, we also found no statistically significant differences in the deprivation index.

Multimorbidity was presented by 71% of our patients, associated with an average of 3.21 chronic diseases. According to this finding, patients with vitiligo should be monitored to prevent these comorbidities or diagnose them early.

According to our findings, the comorbidities more strongly, and the most frequently, associated with vitiligo were thyroid disorders. Conditions such as autoimmune thyroiditis, hypothyroidism, hyperthyroidism, Hashimoto’s thyroiditis, and Graves’ disease show significantly higher prevalence in individuals with vitiligo compared to the general population [17,26]. This connection highlights the shared autoimmune basis of these diseases, where the dysregulation of the immune system leads to the production of autoantibodies targeting melanocytes and thyroid tissue. The presence of Hashimoto’s thyroiditis or hyperthyroidism can influence the activity of vitiligo. Elevated levels of anti-thyroid antibodies are frequently associated with increased disease activity. On the other hand, correcting thyroid dysfunctions could improve the stability of vitiligo in certain patients [27]. Studies have reported that up to 37% of vitiligo patients exhibit thyroid dysfunction, emphasizing the importance of routine screening for thyroid function in this population to enable the early detection and management of associated complications [5,17,28].

In addition, a systematic review and meta-analysis by Lee et al. in 2023 [17] described that patients with vitiligo were more likely to have autoimmune thyroiditis (OR = 10.39, 95% CI = 2.43–44.40), hypothyroidism (OR = 5.54, 95% CI = 3.36–9.13), hyperthyroidism (OR = 4.68, 95% CI = 1.75–12.50), Grave’s disease (OR = 2.93, 95% CI = 2.62–3.28), Hashimoto’s thyroiditis (OR = 2.12, 95% CI = 1.92–2.34), and thyroid cancer (OR = 1.13, 95% CI = 1.02–1.24).

Ear and sense organ disorders were the second most frequent comorbidity in our sample, and were also statistically associated. Melanocytes are abundant in the uveal tract and in the pigment epithelium of the retina. A higher prevalence of hypopigmented spots has been found in both locations, as well as decreased visual acuity, dry eye syndrome, normotensive glaucoma, and chronic progressive neuropathy. Melanocytes are also distributed in the membranous labyrinth of the inner ear, and sensorineural deafness has been observed in some patients [17,18,29,30].

The meta-analysis by Lee et al. [17] showed that glaucoma (OR = 1.31, 95% CI = 1.27–1.35), cataracts (OR = 1.30, 95% CI = 1.27–1.32), iris changes (OR = 1.25, 95% CI = 1.17–1.34), and retinal pigment epithelium changes (OR = 1.19, 95% CI = 1.16–1.22) were significantly more prevalent in patients with vitiligo. With regard to hearing abnormalities, a statistically significant association with sensorineural hearing loss (OR = 2.43, 95% CI = 1.50–3.93) was observed compared to controls without vitiligo.

In addition, the study by Genedy et al. [29] evaluated 40 patients with vitiligo and 20 healthy controls. The results showed a higher prevalence of hearing loss and ocular abnormalities in vitiligo patients compared to controls, although no significant differences in visual acuity were found between the groups.

Regarding the possible common mechanisms, the destruction of melanocytes, which are present in the retinal pigment epithelium, choroid, and cochlear structures, is a central mechanism linking vitiligo to conditions such as uveitis, glaucoma, and sensorineural hearing loss [31]. Oxidative stress plays a pivotal role, leading to cellular damage in these organs [32,33]. Autoimmune inflammation, driven by proinflammatory cytokines like IFN-γ, TNF-α, and IL-1β, exacerbates melanocyte dysfunction and tissue injury in the eyes and ears, further reinforcing the connection. Additionally, the systemic inflammatory environment generated in vitiligo, is characterized by elevated levels of chemokines like CXCL10 [12,34]. These abnormalities could indicate heightened inflammatory and autoimmune activity in patients with vitiligo, correlating with a more severe or active course of the disease.

Other inflammatory skin conditions were the second group of comorbidities most strongly associated with vitiligo in the present study. The most commonly reported concomitant dermatological conditions in patients with vitiligo are atopic dermatitis, psoriasis, and alopecia areata [3,17]. In the meta-analysis by Lee et al. [17], a significant association was revealed between vitiligo and psoriasis (OR = 3.22; 95% CI = 3.07–3.37) as well as atopic dermatitis (OR = 2.45; 95% CI = 2.38–2.52). Additionally, in the study conducted by Ezzedine et al. [35], it was found that the most frequent dermatological diseases among these individuals included atopic dermatitis (3.1% vs. 1.1%), psoriasis (2.7% vs. 0.6%), and linear morphea (1.5% vs. 0.1%). Furthermore, in the study by Rios-Duarte et al. in 2023 [28], cutaneous disorders with the largest effect sizes were alopecia areata and systemic sclerosis.

Both vitiligo and psoriasis are driven by proinflammatory cytokines, with IFN-γ and TNF-α playing central roles in vitiligo, while IL-17, IL-23, and TNF-α are key in psoriasis, exacerbating damage through ROS in both conditions. Autoimmune mechanisms in vitiligo, mediated by cytotoxic CD8+ T cells, and dysregulated immune responses in psoriasis highlight the interaction between T cells and target cells in the skin. Additionally, both conditions exhibit the *Koebner phenomenon* [36]. On the other hand, 23 co-expressed genes have been identified between vitiligo and atopic dermatitis. Additionally, the chronic inflammatory state of atopic dermatitis can induce the *Koebner phenomenon* in vitiligo. Additionally, both conditions exhibit the activation of common pathways, such as Th17 cells, which can promote the activation of the Th2 pathway implicated in both diseases. In the case of alopecia areata, it has been suggested that the immune response driven by IFN-γ is the main driver of the pathogenesis of both diseases [37,38].

Our study also found a high prevalence of connective tissue diseases among patients with vitiligo, with a strong association between vitiligo and these conditions. Systemic lupus erythematosus (SLE), rheumatoid arthritis (RA), Sjögren’s syndrome, and systemic sclerosis have been reported at higher prevalence in patients with vitiligo compared to the general population [39]. In this regard, the study conducted by Lee et al. [17] indicated that systemic sclerosis (OR = 5.06, 95% CI = 2.89–8.87), discoid lupus erythematosus (OR = 2.54, 95% CI = 1.74–3.72), Sjögren’s syndrome (OR = 2.50, 95% CI = 1.98–3.16), SLE (OR = 1.96, 95% CI = 1.52–2.52), and RA (OR = 1.82, 95% CI = 1.55–2.15) were significantly more prevalent in patients with vitiligo. A nationwide cross-sectional study by Rios-Duarte et al. in 2023 [28] concluded that the most frequent autoimmune disorders in patients with vitiligo were type 1 diabetes, RA, SLE, autoimmune thyroiditis, Addison’s disease, and systemic sclerosis. Other comorbidities with the largest effect sizes were primary sclerosing cholangitis, pernicious anemia, Addison’s disease, and autoimmune thyroiditis.

The presence of overlapping genetic factors supports the autoimmune basis of this association. PTPN22 is associated with various autoimmune diseases, including rheumatoid arthritis and systemic lupus erythematosus, due to its role in T-cell signaling dysregulation [39]. Variants in HLA genes, particularly HLA-DQB1 and HLA-DRB1, are strongly linked to increased susceptibility to vitiligo [40]. Additionally, FOXP3 and IKZF4, critical for regulatory T-cell function and immune tolerance, contribute to the suppression of autoimmunity [41]. The IFIH1 gene, which regulates type I interferon production, plays a significant role in innate immune responses [42], while NLRP1 contributes to inflammatory processes through its involvement in the inflammasome [43]. Furthermore, XBP1, a regulator of HLA class II expression and stress responses, connects endoplasmic reticulum stress to autoimmune pathways in vitiligo [44]. Furthermore, CXCL12 and CCL5 contribute to the recruitment and activation of immune cells, such as T lymphocytes, in affected skin areas, exacerbating inflammation and melanocyte destruction. In connective tissue diseases like SLE and RA, CXCL12 facilitates the migration of immune cells to inflammatory sites, while CCL5 amplifies the recruitment of T cells and monocytes, promoting chronic inflammation and tissue damage. These shared inflammatory pathways highlight the overlapping mechanisms in vitiligo and connective tissue diseases, suggesting that the dysregulation of CXCL12 and CCL5 may serve as a common link, driving autoimmunity and sustained immune activation in these conditions [45]. Vitiligo shares inflammatory and oxidative stress mechanisms with connective tissue diseases such as SLE, RA, and systemic sclerosis, leading to mutual influence on their progression. Systemic inflammation and the imbalance of ROS in vitiligo can exacerbate tissue damage in connective tissue diseases, such as endothelial damage and fibrosis in systemic sclerosis or the activation of inflammatory pathways in lupus [6,7].

Although patients with vitiligo were more likely to present cardiac arrhythmias in our study, no connection between the two diseases has been identified in the literature. Stevens et al. [46] described a case of giant-cell myocarditis in a patient with vitiligo, showing that the association found between these two diseases suggests an autoimmune cause of these diseases.

Regarding the association found between vitiligo and urinary and lower respiratory tract infections in the present study, no association between these conditions has been described in the literature. However, there is some evidence suggesting that certain infections or infection-related factors may play a role in triggering or exacerbating flares in autoimmune diseases in susceptible individuals [47]. In the article by Consany et al. [47], infections are highlighted as a frequent complication in patients with systemic autoimmune diseases, with a prevalence of 46.9%, primarily involving non-opportunistic infections. The most common types include respiratory infections (48.6%), urinary tract infections (31.7%), and skin and soft-tissue infections (17.6%). Risk factors identified include the use of immunosuppressive treatments such as methotrexate, mycophenolate, corticosteroids, and biological therapies, as well as active disease and combination treatments. To explore this potential connection, it would be advisable to directly investigate the influence of infections on the development or worsening of vitiligo.

Although anxiety and depression appear first and second, respectively, among the comorbidities of our patients, no statistically significant association with vitiligo was found. The psychological and emotional consequences of vitiligo have a great impact on the quality of life of these patients, especially in those with high skin phototypes, and can lead to low self-esteem, social isolation, and the presence of associated psychiatric comorbidities [3,17,48,49]. In a systemic review [50], the prevalence of suicidal ideation ranged from 6% to 25%. The same findings were found in a retrospective cross-sectional analysis with a rate of depression among patients with vitiligo of 6.8%. Female sex was significantly more associated with depression. Furthermore, Ezzedine et al. [35] observed higher incidence of psychiatric illness in patients with vitiligo than in healthy individuals (28.4% vs. 22.8%), with the most frequent illnesses being anxiety (14.3%), sleep disturbance (9.1%), and depression (8.0%). Depression and anxiety can exacerbate the course of vitiligo by increasing stress levels, which activate inflammatory mechanisms and oxidative stress. In turn, the progression of vitiligo can intensify mental health issues, creating a harmful cycle.

A limitation of this study is the cross-sectional and retrospective design, which precludes the assessment of longitudinal trends within the population. In addition, the absence of certain variables—such as lifestyle factors or laboratory parameters—may limit our ability to fully interpret the results. Another significant limitation is the variability in the quality and standardization of the clinical data extracted from the EHRs, which were not initially designed for research purposes. This non-standardization could lead to potential overdiagnosis or underdiagnosis of some chronic diseases, as well as inconsistencies in data recording and coding across different healthcare settings. Despite these limitations, efforts were made to mitigate these biases by carefully validating and contextualizing our findings with the existing literature.

A key strength of our study is that it was conducted using a population-based cohort, covering 98% of the reference population. It is also worth noting that this study comprehensively examined all chronic conditions recorded in the patients’ EHRs by healthcare professionals, rather than focusing solely on the most common or significant diseases. Additionally, the data within the EpiChron Cohort undergo regular quality control assessments to ensure their accuracy and reliability for research purposes.

## 5. Conclusions

Our study revealed the presence of various clinically significant comorbidities in patients with vitiligo, highlighting the importance of understanding its broader systemic implications. According to our results, all patients with vitiligo should have their thyroid function tested. Ophthalmological and auditory evaluations should be included, particularly in patients with advanced disease, long duration, or related symptoms. A complete dermatological exploration is needed in order to exclude other dermatoses commonly associated with vitiligo. In cases where clinical suspicion for other autoimmune diseases arises, further diagnostic workup should be performed. These findings underscore the importance of clinical management strategies focused on the early detection of specific comorbidities. Looking ahead, the establishment of a diagnostic and therapeutic management algorithm tailored to the comorbidities of patients with vitiligo could significantly enhance patient care and outcomes.

## Figures and Tables

**Table 1:** Detailed list of 26 socio-economic indicators analyzed [21].

Indicator Category	Indicator
Education	% Insufficient Education
Education	% Insufficient Education in Youth
Education	% Insufficient Education (16–64 years)
Education	% Insufficient Education in Foreign Population
Family Structure	% Single-Mother Families
Family Structure	% Single-Father Families
Family Structure	% Single-Person Households (65+ years)
Demographics	% Aging Population (65+ years)
Demographics	% Aging Population (85+ years)
Demographics	% Foreign Population
Employment and Work	% Manual Workers (16+ years)
Employment and Work	% Unemployment
Employment and Work	% Temporary Workers
Housing	% Houses without Heating
Housing	% Houses without Bathroom
Housing	% Houses without Shower/Bath
Housing	% Houses without Internet
Housing	% Houses < 45 square meters
Housing	% Houses with Outstanding Payments
Housing	% Houses Rented
Buildings	% Non-Accessible Buildings
Buildings	% Buildings without Elevators
Buildings	% Buildings in Poor Condition
Buildings	% Non-Accessible Houses
Buildings	% Houses in Buildings without Elevators
Buildings	% Houses in Buildings in Poor Condition

**Table 2:** Socio-demographic and clinical characteristics of patients with vitiligo in the EpiChron Cohort in 2019.

Characteristics	Men	Women	Total
N (%)	95 (43.58)	123 (56.42)	218 (100)
Mean age, years (SD ^1^)	42.45 (22.97)	45.15 (19.85)	43.97 (21.26)
Age group, years (*n*, %)			
0–17	23 (24.21)	13 (10.57)	36 (16.51)
18–44	23 (24.21)	53 (43.09)	76 (34.86)
45–64	31 (32.63)	36 (29.27)	67 (30.73)
≥65	18 (18.95)	21 (17.07)	39 (17.89)
Nationality (*n*, %)			
Spain	71 (74.74)	94 (76.42)	165 (75.69)
Eastern Europe	4 (4.21)	8 (6.50)	12 (5.50)
Asia	0 (0)	1 (0.81)	1 (0.46)
North Africa	4 (4.21)	3 (2.44)	7 (3.21)
Sub-Saharan Africa	1 (1.05)	1 (0.81)	2 (0.92)
Latin America	14 (14.74)	16 (13.01)	30 (13.76)
EU and North America	1 (1.05)	0 (0)	1 (0.46)
Area of residence			
Urban ^2^ (*n*, %)	58 (61.05)	70 (56.91)	128 (58.72)
Deprivation index ^3^ (*n*, %)			
Q_1_	13 (13.68)	37 (30.08)	50 (22.94)
Q_2_	30 (31.58)	29 (23.58)	59 (27.06)
Q_3_	24 (25.26)	18 (14.63)	42 (19.27)
Q_4_	28 (29.47)	39 (31.71)	67 (30.73)
Number of chronic diseases (mean, SD)	2.75 (1.92)	3.56 (2.68)	3.21 (2.41)
Multimorbidity, yes (*n*, %)	62 (65.26)	94 (76.42)	156 (71.56)

^1^ standard deviation; ^2^ versus rural; ^3^ deprivation index of the residence area according to 26 socio-economic indicators and categorized from least (Q1) to most (Q4) deprived.

**Table 3:** Likelihood of presenting vitiligo based on sex, age, nationality, area of residence, and deprivation index.

Variable	Crude OR ^1^	*p*-Value	Adjusted OR ^2^	*p*-Value
Sex				
Men	ref.			
Woman	1.14 (0.87–1.48)	0.349		
Age group (years)				
0–17	ref.			
18–44	0.93 (0.63–1.38)	0.724		
45–64	0.73 (0.49–1.09)	0.129		
≥65	0.48 (0.30–0.76)	0.002		
Nationality				
Spain	ref.		ref.	
Sub-Saharan Africa	1.07 (0.27–4.32)	0.924	1.01 (0.25–4.08)	0.987
Asia	0.99 (0.14–7.09)	0.994	0.91 (0.13–6.52)	0.927
Eastern Europe	1.56 (0.87–2.80)	0.139	1.45 (0.81–2.61)	0.213
Latin America	3.30 (2.23–4.87)	<0.001	3.06 (2.07–4.53)	<0.001
North Africa	2.04 (0.96–4.36)	0.064	1.92 (0.90–4.10)	0.091
EU and North America	0.60 (0.08–4.27)	0.607	0.60 (0.08–4.26)	0.606
Area of residence				
Urban	ref.		ref.	
Rural	1.07 (0.82–1.41)	0.597	1.08 (0.83–1.42)	0.552
Deprivation index ^3^ (*n*, %)				
Q_1_	ref.		ref.	
Q_2_	1.27 (0.87–1.86)	0.208	1.29 (0.88–1.88)	0.188
Q_3_	1.08 (0.72–1.62)	0.720	1.10 (0.73–1.66)	0.650
Q_4_	1.28 (0.89–1.85)	0.181	1.30 (0.90–1.88)	0.160

^1^ odds ratio; ^2^ adjusted odds ratios for sex and age; ^3^ deprivation index of the residence area according to 26 socio-economic indicators and categorized from least (Q1) to most (Q4) deprived.

**Table 4:** Prevalence of chronic comorbidities in patients with vitiligo in EpiChron Cohort in 2019 (*n* = 218) and likelihood of vitiligo depending on comorbidities. The table shows the comorbidities in order of prevalence.

Comorbidity	Prevalence	Crude OR	Adjusted OR ^1^	*p*-Value ^2^
	*n* (%)	(95% CI)	(95% CI)	
Disorders of lipid metabolism	60 (27.5)	0.86 (0.64–1.15)	1.11 (0.80–1.53)	0.532
Thyroid disorders	55 (25.2)	2.58 (1.90–3.50)	3.01 (2.18–4.15)	<0.001 *
Hypertension	50 (22.9)	0.74 (0.54–1.02)	1.12 (0.76–1.63)	0.569
Other nutritional, endocrine; and metabolic disorders	41 (18.8)	0.97 (0.69–1.36)	1.18 (0.83–1.68)	0.356
Spondylosis; intervertebral disk disorders; other back problems	39 (17.9)	1.26 (0.89–1.78)	1.42 (1.00–2.02)	0.051
Other ear and sense organ disorders	35 (16.1)	1.41 (0.98–2.02)	1.54 (1.07–2.22)	0.020 *
Anxiety disorders	29 (13.3)	0.91 (0.61–1.34)	0.92 (0.62–1.36)	0.660
Blindness and vision defects	28 (12.8)	1.47 (0.99–2.19)	1.44 (0.97–2.15)	0.070
Menstrual disorders	24 (11.0)	1.39 (0.91–2.13)	1.23 (0.78–1.94)	0.364
Other inflammatory conditions of skin	24 (11.0)	2.15 (1.41–3.29)	2.21 (1.45–3.38)	<0.001 *
Osteoarthritis	23 (10.6)	0.95 (0.62–1.47)	1.50 (0.93–2.41)	0.093
Other upper respiratory disease	22 (10.1)	0.79 (0.51–1.23)	0.75 (0.48–1.16)	0.196
Other connective tissue disease	22 (10.1)	1.77 (1.14–2.76)	1.84 (1.19–2.87)	0.007 *
Allergic reactions	22 (10.1)	1.05 (0.67–1.63)	0.91 (0.58–1.43)	0.674
Depression and mood disorders	22 (10.1)	0.78 (0.50–1.21)	0.88 (0.56–1.37)	0.563
Headache, including migraine	21 (9.6)	0.97 (0.62–1.52)	0.89 (0.57–1.40)	0.624
Other lower respiratory disease	20 (9.2)	1.78 (1.12–2.82)	1.78 (1.12–2.82)	0.014 *
Diabetes mellitus	20 (9.2)	0.96 (0.61–1.53)	1.39 (0.86–2.27)	0.181
Asthma	19 (8.7)	1.23 (0.77–1.98)	1.16 (0.72–1.85)	0.546
Urinary tract infections	19 (8.7)	1.58 (0.98–2.52)	1.69 (1.05–2.73)	0.032 *
Neoplasms	16 (7.3)	1.25 (0.75–2.08)	1.49 (0.89–2.49)	0.131
Genitourinary symptoms and ill-defined conditions	15 (6.9)	0.91 (0.54–1.53)	1.32 (0.76–2.29)	0.323
Obesity	15 (6.9)	0.69 (0.41–1.17)	0.76 (0.45–1.29)	0.316
Cardiac dysrhythmias	14 (6.4)	1.26 (0.74–2.17)	1.84 (1.05–3.22)	0.034 *

^1^ odds ratios adjusted by sex and age; ^2^ *p*-values for the adjusted OR; * *p* < 0.05.

## Data Availability

The data used in this study cannot be publicly shared because of restrictions imposed by the Aragon Health Sciences Institute (IACS) and asserted by the Clinical Research Ethics Committee of Aragon (CEICA, ceica@aragon.es). The authors can establish future collaborations with other groups based on the same data. Potential collaborations should be addressed to the Principal Investigator of the EpiChron Group, Antonio Gimeno-Miguel, agimenomi.iacs@aragon.es.
